# Influence of different antioxidant agents on the microtensile bond strength of restored teeth after bleaching

**DOI:** 10.1080/07853890.2021.1897355

**Published:** 2021-09-28

**Authors:** Sofia Lobo, Inês Caetano Santos, António H. S. Delgado, Luís Proença, José João Mendes

**Affiliations:** a Instituto Universitário Egas Moniz (IUEM), Egas Moniz Cooperativa de Ensino Superior, Caparica, Portugal; b Centro de Investigação Interdisciplinar Egas Moniz (CiiEM), Egas Moniz Cooperativa de Ensino Superior, Caparica, Portugal; Instituto Universitário Egas Moniz, Caparica, Portugal

## Abstract

**Introduction:**

Due to an increase in patient awareness and search for aesthetic treatments, dental bleaching is a frequent and safe procedure in clinical practice for the removal of stains [1]. Bleaching agents are known to adversely affect the bond strength between resin composite and tooth surface, when adhesive procedures are performed immediately after tooth bleaching [2]. The reduction in bond strength is related to the presence of residual oxygen, a sub product of hydrogen peroxide that remains on the tooth surface and which may interfere with infiltration of the resin in the dentine tubules and inhibit the polymerisation of resin monomers [3]. Antioxidant agents like sodium ascorbate, grape seed extract and green tea may be used as an alternative to delay the restorative procedure due to their potential as reversers of these adverse effects [2]. This study aims to assess the influence of different antioxidant agents on the bond strength of restored bleached teeth.

**Materials and methods:**

The present study was approved by the Ethics Committee of Instituto Universitário Egas Moniz. Fifteen human permanent molars were sectioned into identical halves that were randomly distributed between five groups (*n* = 6): unbleached control group (CG), bleaching + resin composite bonded immediately (G1), bleaching + sodium ascorbate (G2), bleaching + grape seed extract (G3) and bleaching + green tea (G4). G1, G2, G3 and G4 were bleached for 4 h/day for a 7-day period. After bleaching, G1 samples were immediately restored with an adhesive system and a resin composite, in G2 samples a 10% sodium ascorbate gel was applied, in G3 a 5% grape seed extract and in G4 a 5% green tea, all applied for 15 min. After these antioxidants, G2, G3 and G4 were immediately restored. After 24 h, samples were sectioned in order to obtain 1.0 (± 0.3) mm^2^ microspecimens. The microspecimens were tested in a universal testing machine at a speed of 0.5 mm/min. Data were analysed by using a two-way ANOVA, at a significance level of 5%.

**Results:**

G1 group (bleaching only) recorded the lowest mean bond strength value (9.5 (± 1.2) MPa) and it was significantly lower than the control group (CG) (19.3 (± 2.7) MPa) (*p* = .001). Groups in which sodium ascorbate (G2) (19.2 (± 1.3) MPa), grape seed extract (G3) (16.5 (± 0.8) MPa) and green tea (G4) (16.7 (± 2.0) MPa) were applied presented significantly higher bond strength values when compared to bleaching only (G1) (*p* < .001). When comparing the antioxidant agents, G2 (10% sodium ascorbate) exhibited significantly higher mean bond strength values when compared to G3 (5% grape seed extract) (*p* = .016).

**Discussion and conclusions:**

Treatment of the enamel surface with antioxidant agents such as sodium ascorbate, grape seed extract and green tea following the bleaching procedure and immediately before the restorative procedure can reverse the compromised bond strength. These alternative strategies are effective and may be used instead of delaying the procedure.
